# Sleep, Fatigue and Quality of Life: A Comparative Analysis among Night Shift Workers with and without Children

**DOI:** 10.1371/journal.pone.0158580

**Published:** 2016-07-08

**Authors:** Silvio Araújo Fernandes-Junior, Francieli Silva Ruiz, Leandro Stetner Antonietti, Sergio Tufik, Marco Túlio de Mello

**Affiliations:** 1 Department of Psychobiology, Federal University, São Paulo, Brazil; 2 Center for Multidisciplinary Studies in Sleepiness and Accidents (CEMSA), São Paulo, Brazil; Charité—Universitätsmedizin Berlin, GERMANY

## Abstract

**Introduction:**

The reversal of the natural cycle of wakefulness and sleep may cause damage to the health of workers. However, there are few studies evaluating sleep, fatigue and quality of life of night shift workers considering the influence of small children on these variables.

**Aims:**

Evaluate the sleep time, fatigue and quality of life of night shift workers and verify the relationship between these variables with the presence or absence of children in different age groups.

**Methods:**

Were evaluated 78 mens shiftworkers, with or without children. Group 1, workers without children (G1-*NC*), group 2, workers with children pré-school age (G2-*PS*) and group 3, workers with children school age (G3-*S*). The sleep time (ST), sleep efficiency (SE), sleep latency (SL) and maximum time awake (MTA) were recorded by actigraphy. The risk of being fatigued at work was estimated by risk index for fatigue (RIF).

**Results:**

The G1-*NC* showed a longer ST on working days and when evaluated only the first nights shift, after day off (p<0,005). This sample, the age of the children did not influence the sleep time these workers. The MTA on day off was lower in the workers from G2-*PS*. The RIF was lower on G1-*NC* in the first nights shift compared to the other groups.

**Conclusion:**

In this research, workers without children had higher sleep time during the working days. These workers also were less likely to feel fatigued during night work than workers with children, regardless of age these children.

## Introduction

Workers’ health and well-being depend on the interaction of various individual, medical, psychosocial and job-related factors. In most developed and developing countries, such as Brazil, a large number of workers have reduced sleep times, mainly because of work shifts that begin in the early morning hours. This nocturnal work can restrict nighttime sleep and increase the need to sleep during the day [1,2]. These workers, defined as *shift workers*, are more likely to get sick, especially because of their irregular sleep-wake cycles and sleep deprivation [3].

Chronic sleep restriction promotes immunological [4], sexual behavior [5], cardiovascular and metabolic changes [6,7,8]. These workers demonstrate acutely higher risk of fatigue and excessive sleepiness during work hours [9–10]. Social factors may also affect sleep in this population. Social conflicts associated with unconventional work schedules can be a negative factor for the quality of life and sleep among these individuals [11]. However, most studies that explored the social impacts of shift work were performed with female workers [11,12,13]. Some studies, however, have correlated the risks of work injury between men and women with and without children [14]. Nevertheless, few studies have evaluated the impact of the presence of children on sleep and fatigue among male shift workers. In this regard, the different levels of social dependence associated with children’s different ages should restrict the free time for diurnal sleep, thus decreasing sleep quality and increasing the risk for fatigue during work. These factors, if well understood, could help us to adopt strategies to ensure better health and work for this growing population.

The central hypothesis of this study is that night shift workers who live with children at home will have reduced sleep and are at greater risk of feeling fatigued compared with night shift workers on the same schedule who do not have children.

## Aims

The main aims of this study are to evaluate the average sleep time, the risk of fatigue and the quality of life of male night shift workers and to verify the relationship between these variables and the presence or absence of children of different ages.

## Methods

### Participants and sampling

The study was carried out in accordance with the Declaration of *Helsinki’s ethical standards* and was approved by the Research Ethics Committee at the Universidade Federal de São Paulo, Brazil. This research was conducted in a Brazilian company that extracted iron ore. The assessments took place between January and December of 2013.

#### Inclusion criteria

Married men who were 20 to 50 years old and who had worked for more than six months in the same position were considered. The shift workers were employed on the following schedules: day 1: 18:00–06:00 hours; day 2: 18:00–06:00 hours; 48 hours of rest; resumption of the cycle.

#### Exclusion criteria

Individuals who claimed to have any type of sleep disorder were not selected. We excluded any subject with a body mass index (BMI) higher than 30 kg/m^2^ or under 18 kg/m^2^. Subjects who classified themselves as extreme morning types or extreme evening types and those who had children older than 18 years old were also excluded.

#### Sample composition

The subjects were divided into three (3) groups. Group 1, workers without children (G1 no children or G1-NC), group 2, workers with children preschool age (between zero (0) and five (5) years old) (G2-PS), and group 3, workers with school-aged children, between six (6) and seventeen (17) years old (G3-S).

**Fig 1:**
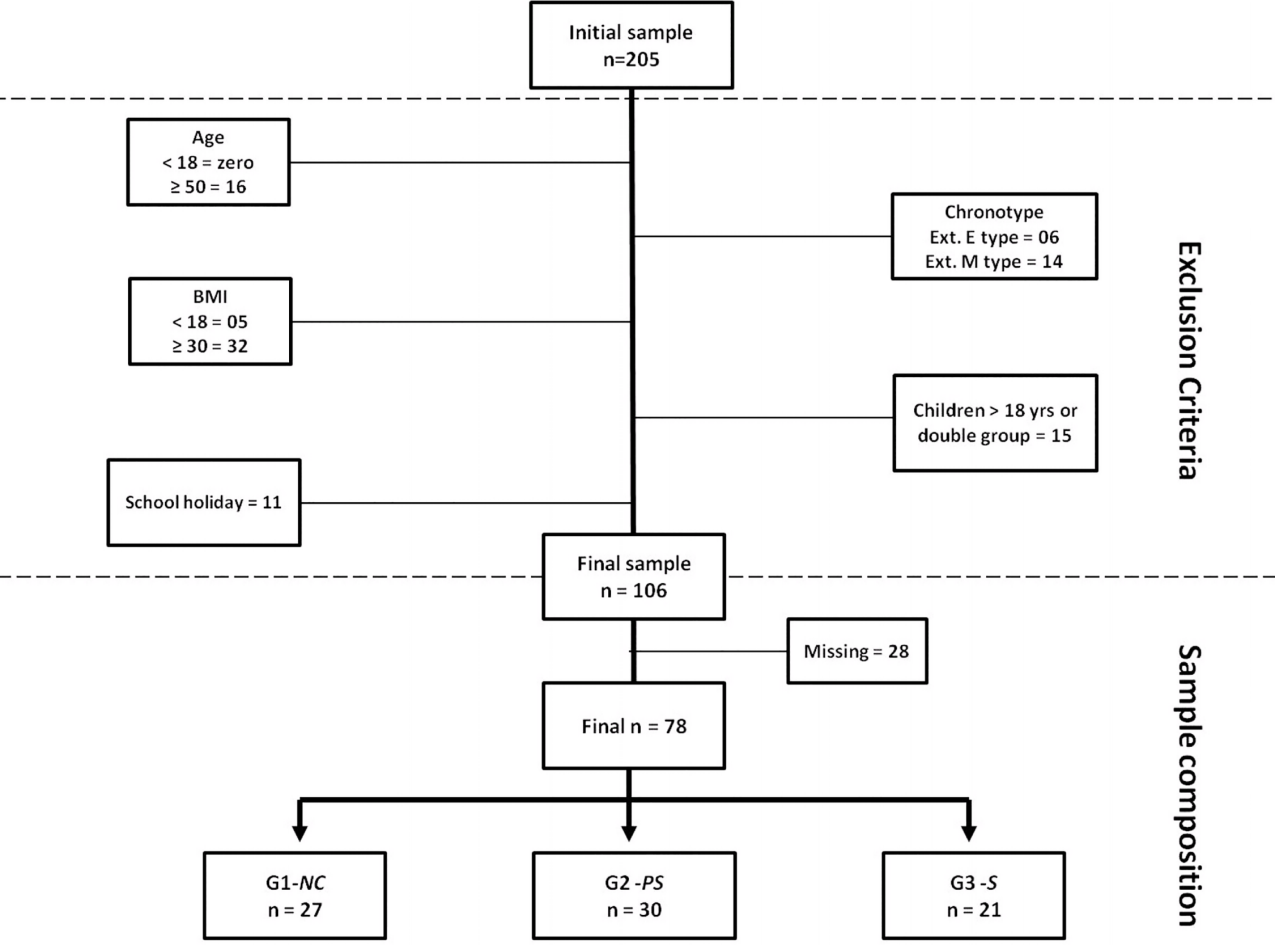
Exclusion criteria of the study and sample composition. Ext. E type = extreme evening type; Ext. M type = extreme morning type; BMI = body mass index; G1-*NC* = group 1, workers without children; G2-*PS* = group 2, workers with preschool age children; G3-*S =* workers with school-age children.

The study also did not include volunteers who had two or more children of different ages, which would have classified them into different groups (double group). Evaluations of subjects who had been evaluated during their children’s school holidays in January, June, July and December were disregarded (Fig 1).

### Experimental Design

#### First step

Individuals completed the General Identification Record (GIR). These questionnaires assessed marital status, number and age of children, and job function. To ascertain their chronotypes, the subjects answered 19 questions on a questionnaire that was developed by Horne and Ostberg [15] (the *Morning-Evening Questionnaire*–MEQ) and that had been translated into Portuguese and previously validated [16].

The Portuguese language version of the Epworth Sleepiness Scale (ESS) was used [17] to assess sleepiness during daily activities [18]. In this protocol, the validated Portuguese version of the SF-36 was used to analyze the impact of shift work on quality of life [19]. All of the assessments occurred between 9 p.m. and 11 p.m. on the first night shift after a day off.

#### Second step

Sleep was recorded using an actigraphy monitor (*model Actiwatch-64; Philips/Respironics*, *Andover*, *Mass*., *USA*). Actigraphy is considered a valid procedure for measuring motor activity patterns and for classifying possible circadian disorders [20]. The use of the actigraphy monitor allowed for evaluating sleep time (ST), sleep efficiency (SE), sleep latency (SL) and maximum time awake (MTA) over the 10 days of observation. Sleep data on work nights and days off were evaluated separately, as were the data from the first vs. the last night’s work. The risk of being fatigued at work was estimated using a risk index for fatigue (RIF) that was proposed by international agencies to control flight safety and manage fatigue risks [21]. The calculation of RIF is based on the conceptual foundation theory of homeostatic and circadian factors that control sleep [22] and on previous studies on regulating alertness and human fatigue [23,24,25]. For the RIF, we considered the sleep episodes of the last 24 and 48 hours, beyond the wake time at the initial and final moments of each night shift. The initial moments (M1) and final moments (M2) in this study represent the start (6 p.m.) and end (6 a.m.) times of the job.

### Statistical Analyses

The statistical software (*version 17*.*0- SPSS*, *Inc*, *Chicago*, *IL*, *USA*) was used for all calculation. The normality of the data was assessed with the Kolmogorov-Smirnov test. The descriptive statistics consisted of calculating the mean ± standard deviation for the quantitative data. The between-group quantitative data comparisons were performed using a *one-way* ANOVA. The Bonferroni post-test was applied.

Homoscedasticity was tested using the Levene test, and, when necessary, the Brown-Forsythe correction was used. A repeated-measures ANOVA (time × group) was used in the comparisons between groups involving different working times (e.g., fatigue). The data were adjusted using an ANCOVA whenever the waking time that preceded the workday would have influenced the outcome of the comparison between groups.

Analysis of covariance (ANCOVA) was used in cases in which age could have potentially interfered with the results. In these cases, multiple comparisons were performed using the confidence interval settings (*Sidak*). The descriptive statistics for the qualitative data consisted of calculating the absolute and relative frequencies, and comparisons between groups were performed using the *Pearson* ***x^2^*** *test*. The effect size (ES) and power of the test were calculated for those analyses. The value of α = 5% was considered to be statistically significant.

## Results

### Characteristics of the sample

Initially, 205 workers were evaluated in step 1. After the exclusion criteria were applied, 106 were selected for the next phase. Throughout the 10 days of monitoring by actigraphy, 28 subjects were discarded for technical equipment problems and/or incorrect use of the actigraph (missing data). Thus, the final sample for analysis comprised 78 subjects divided into 3 groups: G1-*NC* (n = 27), G2-*PS* (n = 30) and G3-*S* (n = 21) (Fig 1). The average age was 31.6 ± 6 years. The body mass index, circadian preference (chronotype), sleepiness scale scores and job functions (function) were compared and are shown in Table 1.

**Table 1:** General characteristics of the sample and sleepiness scale scores.

		G1-*NC* (n = 27)	G2-*PS* (n = 30)	G3-*S* (n = 21)	Total (n = 78)	Chi^2^	p
	**Age (years)**	26.85 ± 4.33	32.4 ± 5.83 ^a^	36.81 ± 4.58 ^a^^.^ ^b^	31.67 ± 6.33	23.737	<0.001*
**BMI**	*Normal weight*	11 (40. 74%)	12 (40%)	5 (23. 81%)	28 (35. 9%)	1.828	0.401
	*Overweight*	16 (59. 26%)	18 (60%)	16 (76. 19%)	50 (64. 1%)		
**MEQ**	*Morning types*	3 (11.11%) ^b^	13 (44. 83%)	15 (71.43%) ^a^	31 (40. 26%)	18.666	0.001*
	*Intermediate*	22 (81.48%) ^a^	15 (51. 72%)	6 (28.57%) ^b^	43 (55. 84%)		
	*Evening types*	2 (7. 41%)	1 (3. 45%)	0 (0%)	3 (3. 9%)		
**Type work**	*M*.*operator*	15 (55.5%)	15 (50%)	10 (47.6%)	40 (51.28%)	3.700	0.717
	*Mechanic*	06 (22.2%)	08 (26.6%)	08 (38.0%)	22 (28.2%)		
	*Electrician*	05 (18.5%)	05 (16.6%)	01 (4.76%)	11 (14.1%)		
	*Welder*	01 (3.7%)	02 (6.6%)	02 (9.52%)	05 (6.4%)		
**ESS**	*Drowsy*	14 (51.85%) ^b^	21 (70%)	18 (85.71%) ^a^	53 (67.95%)	6.314	0.043*
	*Not drowsy*	13 (48.15%) ^a^	9 (30%)	3 (14.29%) ^b^	25 (32.05%)		

Age: mean ± standard deviation (ANOVA) one-way with Bonferroni post hoc. Other data: Pearson X^2^ test. BMI = body mass index; MEQ = morningness and eveningness questionnaire; M.operator = machine operator; ESS = Epworth Sleepiness Scale; Chi^2^ = chi-square

a = adjusted residual > 1.96

b = adjusted residual < -1.96.

* p< 0.05.

The average age of the workers was significantly different between all groups. The G1-*NC* subjects were younger than the G3-*S* workers. G1-*NC* showed a higher concentration of the intermediate chronotype, and G3-*S* had a higher concentration of morning types. Considering BMI, the sample was homogeneous. Similarly, participants’ job functions were distributed homogeneously (Table 1). In groups 1 and 2, 58.8% (n = 30) had 1 child, 37.3% (n = 19) had 2 children and 3.9% (n = 2) had 3 children. No worker had 4 or more children.

#### Variables of Sleep and Wakefulness

G3-*S* had the highest percentage of individuals who reported not feeling sleepy (Table 1).

ST on working days was significantly different from ST on days off (210.16 ± 70.19 mins/working day *versus* 377.31 ± 54.6 mins/day off (p<0,001)). Moreover, ST was greater in G1-*NC* on working days, both on average work days and on the average first night of work after a day off.

When ST was evaluated only on the last night of work before a day off, G1-*NC* showed more ST only in relation to G3-*S*. ST on days off showed no differences between groups, and no differences in relation to sleep efficiency (SE) were observed. Regarding SL, there was a significant difference only on the first working day. G2-*PS* showed shorter latency compared with the G1-*NC* (Table 2) workers. This study observed less MTA on the days off of workers in G2-*PS* in relation to the G1-*NC* workers. Regarding MTA on work days, it was observed that G3-*S* showed significantly higher values in relation to G1-*NC* and G2-*PS* (Table 2).

**Table 2:** Parameters of sleep and wakefulness.

		G1-*NC* (n = 27)	G2-*PS* (n = 30)	G3-*S* (n = 21)	Total (n = 78)	F	p	PES	Power	
**ST (mins)**	Days off	379.62 ± 50.13	386.02 ± 61.49	361.9 ± 48.58	377.31 ± 54.6	1.250	0.292	0.032		0.264
	Work	262.86 ± 71.12	187.36 ± 57.24 ^a^	174.98 ± 42.54 ^a^	210.16 ± 70.19	17.539	<0.001*	0.307		1.000
	1^st^ night shift	283.61 ± 84.12	174.14 ± 82.09 ^a^	165.07 ± 59.69 ^a^	209.59 ± 93.83	18.909	<0.001*	0.335		1.000
	Last night shift	241.93 ± 88.43	199.97 ± 69.34	187.68 ± 57.04 ^a^	211.18 ± 76.34	3.760	0.028*	0.091		0.670
**SE (%)**	Days off	80.42 ± 6.54	81.03 ± 6.18	79.67 ± 7.11	80.45 ± 6.5	0.268	0.766	0.007		0.091
	Work	79.4 ± 6.13	79.92 ± 6.87	77.14 ± 6.52	78.99 ± 6.54	1.203	0.306	0.031		0.255
	1^st^ night shift	77.49 ± 8.59	79.46 ± 8.92	74.92 ± 7.96	77.56 ± 8.64	1.740	0.183	0.044		0.354
	Last night shift ^†^	79.78 ± 7.32	81.54 ± 5.53	78.28 ± 9.35	80.05 ± 7.35	1.145	0.326	0.032		0.265
**SL (mins)**	Days off^†^	10.71 ± 7.67	9.69 ± 8.64	7.28 ± 3.64	9.4 ± 7.3	1.525	0.225	0.035		0.284
	Work ^†^	9.79 ± 7.12	6.27 ± 5.67	7.54 ± 4.1	7.83 ± 5.99	2.725	0.073	0.064		0.500
	1^st^ night shift	10.69 ± 8.64	5.59 ± 6.2 ^a^	9.85 ± 6.92	8.5 ± 7.59	3.937	0.024*	0.095		0.692
	Last night shift	7.14 ± 6.92	5.62 ± 5.24	5.93 ± 5.94	6.23 ± 6.01	0.484	0.618	0.013		0.127
**MTA (mins)**	Days off	1099.56 ± 173.63	922.12 ± 155.78 ^a^	1013.62 ± 194.32	1008.18 ± 186.95	7.495	0.001*	0.167		0.935
	Work	1165.26 ± 184.84	1163.82 ± 213.92	1312.22 ± 237.95 ^a^^.^ ^b^	1204.27 ± 218.69	3.751	0.028*	0.091		0.669

Mean ± standard deviation (ANOVA) one-way with Bonferroni post hoc.

† = Brown- Forsythe correction

F = ANOVA; PES = Partial Eta Squared for effect size

a = p<0.05 compared with G1-NC

b = p<0.05 compared with G2-PS.

**ST** = sleep time; **SE** = sleep efficiency; **SL** = sleep latency; **MTA** = maximum time awake; **1˚ night shift** = ST, SE, SL only on the first night of work after a day off; **Last night shift** = ST, SE, SL only on the last night of work before a day off.

* p< 0.05.

**Fig 2:**
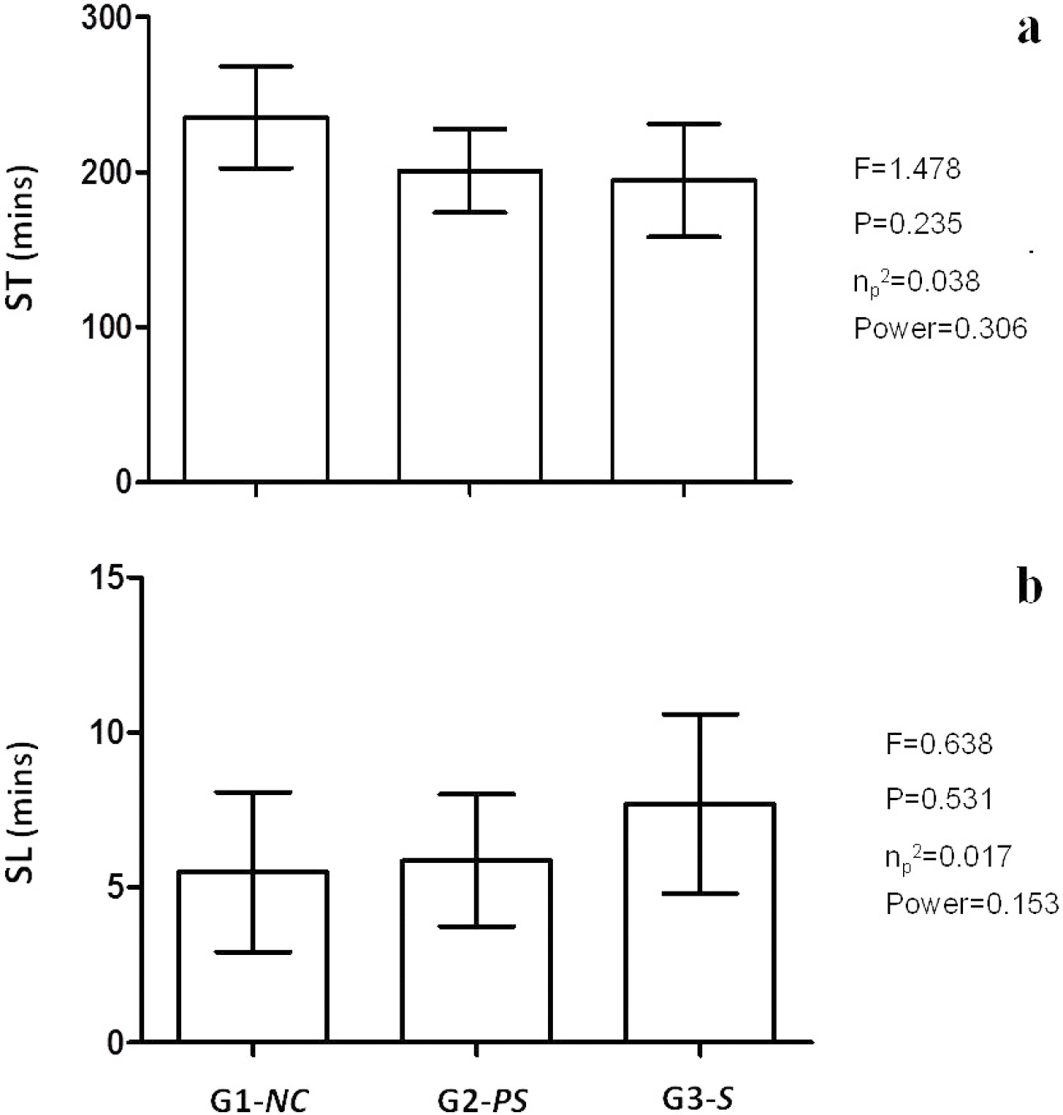
Sleep time (ST) and sleep latency (SL). Mean and standard deviation 95% adjusted for age only on the last day of work. **a)** ST (covariant age p = 0.431); **b)** SL (covariant age p = 0.013*). *ANCOVA one-way with Sidak multiple comparisons*. *F = ANOVA;* PES *= Partial Eta Squared with effect size; Power = observed power*. * *= p<0*.*05*.

There were significant age differences. G1-*NC* contained younger workers, and G3-*S* contained older workers. After analysis of covariance (age factor), the previous results remained except for ST and SL on the last day of work. For ST, the difference disappeared (Fig 2A). For SL, the adjustment did not change the results without the covariant; that is, there remained no significant difference between the groups (Fig 2B).

#### Quality of life

The results of quality of life (QL) are listed in Table 3. There was no significant difference between the groups regarding QL.

**Table 3:** The SF-36 quality of life domains.

	G1-*NC* (n = 27)	G2-*PS* (n = 30)	G3-*S* (n = 21)	Total (n = 78)	F	p	PES	Power
**General score %**	78.97 ± 9.83	78.09 ± 16.13	82.25 ± 8.24	79.52 ± 12.29	0.740	0.481	0.019	0.171
**Functional capacity %**	92.96 ± 11.71	86.17 ± 25.31	93.1 ± 21.65	90.38 ± 20.51	1.032	0.361	0.027	0.224
**Physical aspects %** ^†^	87.96 ± 23.38	92.5 ± 20.92	97.62 ± 7.52	92.31 ± 19.45	1.652	0.200	0.038	0.306
**Pain %**	82.15 ± 20.94	85.73 ± 21.45	84.43 ± 19.04	84.14 ± 20.44	0.217	0.806	0.006	0.083
**General health %**	84.11 ± 13.35	76.03 ± 21.68	84.62 ± 18.6	81.14 ± 18.53	1.899	0.157	0.048	0.383
**Vitality %**	46.67 ± 8.99	42.83 ± 10.23	47.62 ± 5.62	45.45 ± 8.91	2.239	0.114	0.056	0.443
**Social aspects %**	83.8 ± 20.16	85.42 ± 23.23	91.07 ± 19.42	86.38 ± 21.15	0.744	0.478	0.019	0.172
**Emotional aspects %**	93.83 ± 16.11	94.44 ± 19.74	96.83 ± 14.55	94.87 ± 17.05	0.194	0.824	0.005	0.079
**Mental health %**	60.3 ± 5.54	61.6 ± 13.56	62.67 ± 5.84	61.44 ± 9.45	0.373	0.690	0.010	0.108

Mean ± standard deviation (ANOVA) one-way.

† = Brown- Forsythe correction

F = ANOVA; PES = Partial Eta Squared for effect size; Power = observed power.

#### Fatigue

After the assessment of the risk index for fatigue (RIF) on the first working day, there was a significant difference in the group (p<0.001), time (p<0.001) and interaction effects (p = 0.024). All groups had increased RIF scores at the final moment of work in comparison with the initial moment of work (p<0.001). Additionally, G1-*NC* showed lower RIF values in relation to G2-*PS* (p = 0.007) and G3-*S* (p = 0.001), both at entry and in work output (p = 0.002 and p<0.001, respectively). G2-*PS* did not differ from the G3-*S* workers at any moment.

**Fig 3:**
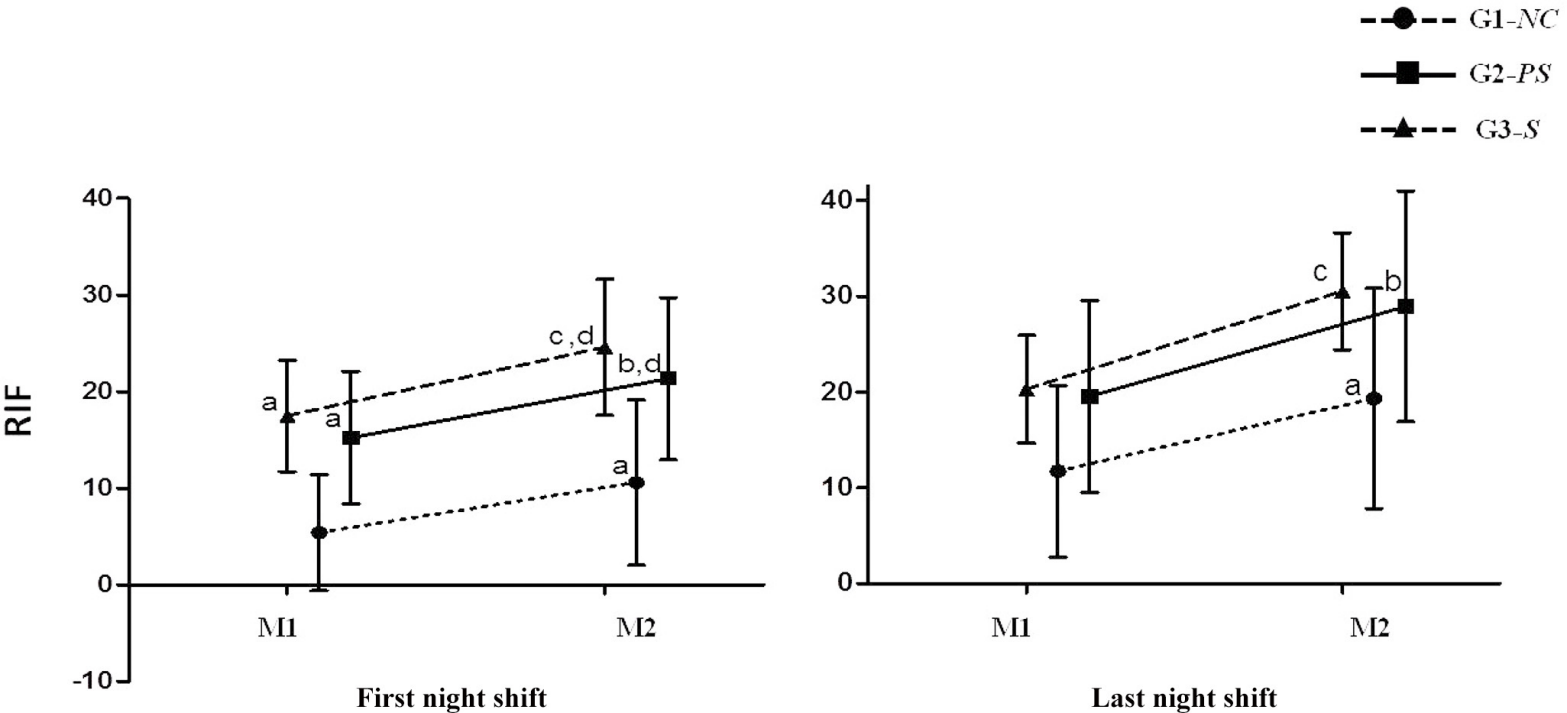
Means and standard deviations of the risk index for fatigue (RIF). (M1) = time of work onset and (M2) time of work completion. **First night shift: a** = p<0.05 in comparison with G1-*NC* at M1; **b** = p<0.05 in comparison with G2-*PS* at M1; **c** = p<0.05 in comparison with G3-*S* at M1 and **d** = p<0.05 in comparison with G1-*NC* at M2. **Last night shift: a** = p<0.05 in comparison with G1-*NC* at M1; **b** = p<0.05 in comparison with G2-*PS* at M1; **c** = p<0.05 in comparison with G3-*S* at M1. *ANOVA for repeated measures with Bonferroni test*.

There was a group effect (p<0.001) for the last night shift, in addition to time (p<0.001) and interaction effects (p<0.001). The *post hoc* test showed that all groups had increased RIF values in the final moment in comparison with the initial moment of work (p<0.001). However, on the last day of work, the groups did not differ (Fig 3).

## Discussion

In this study, we observed that the workers without children (G1-*NC*) showed greater ST on workdays than did workers with children. It was also observed in all groups that there were more episodes of sleep during the night (days off) than during the day (workdays). On days off, there were no statistically significant differences in ST between groups. These results were confirmed even after adjustment for the covariate age. ST showed no differences between the groups with children; therefore, the children’s ages did not influence the subjects’ sleep time.

It is known that daytime sleep does not promote all physiological functions, which leads to poor health consequences, especially through endogenous factors [2,26,27]. However, the decrease in average sleep time may have also been caused by other factors such as social and family factors [11,28]. The present study examined a social factor when considering the presence of children in the homes of the workers.

Some authors have found that the sleep time of workers with children was lower than those workers without children. However, most of these studies were conducted among women, whose sleep was interrupted so they could prepare meals or get their children to school [11,29]. In contrast, the central focus of the present study was to evaluate sleep in male shift workers. A progressive and significant decrease in average sleep duration has been found to be associated with reduced QL [28] in recent decades. The QL questionnaire used in this study reflected employees’ typical complaints (e.g., physical issues, emotional and functional capacity and vitality). In this research, vitality scored the lowest among all items. However, there were no significant differences between groups in these areas. Regarding these workers’ chronotypes, the distribution of this sample was similar to that of a study that evaluated 1165 workers. These researchers observed 48% morning types, 40.3% intermediate types and 2.2% evening types [30]. In our sample of 78 individuals, a similar distribution was observed, with 40.26% morning types, 55.84% intermediate types and 3% evening types.

It is well established in the literature that average sleep time gradually reduces over the course of one’s life [31]. In this study, there was a significant age difference between the groups, with those in G1-*NC* being younger and in G3-*S* being older. These data justified the need to adjust the calculation of the variables related to sleep; however, even after this adjustment, the age factor did not change the results except for those for ST on the last night shift the significant difference disappeared after this control. In this sample, older individuals were classified mostly as morning types and the younger individuals as evening types. These results corroborate the literature, which finds a tendency toward morningness as we age [32,33,34].

It has been demonstrated that the risks for work incidents increase exponentially after successive night shifts [9]. Thus, work on the first and last night shifts is considered to be critical for assessing the relative risk of accidents at work. Therefore, this study separately evaluated ST on the first and last nights of work. Regarding ST on the first night, it was observed that G1-*NC* slept more compared with the other groups. When ST was evaluated only on the last night of work, it was observed that G1-*NC* showed longer ST only in relation to G3-*S*.

The literature demonstrates that long waking hours reduce the capacity and speed of conscious responses and, for this reason, increase the feeling of fatigue by restricting sleep time [35,36]. Dawson and Reid correlated sleep restriction with alcohol intake. The authors showed that when subjects stood for more than 28 hours of wakefulness (from 8:00 until 12:00 the following day), their performance on psychomotor tests was similar to that of individuals with moderate alcohol intoxication [35]. In this study, the overall average MTA on days off was approximately 16 consecutive hours (1008.18 ± 186.95 minutes). In turn, on working days, an average MTA of over 20 hours was observed (1204.27 ± 218.69 minutes). In relation to MTA between groups, there was a significant difference when considering only work days (G1-*NC* = 1165.26 ± 184.84 minutes; G2-*PS* = 1163.82 ± 213.92 minutes and G3-*S* = 1312.22 ± 237.95 minutes). G3-*S* continued to show more time awake. In this case, family commitments and helping children with school activities, among other factors, could have contributed to these workers’ remaining awake longer than workers without children.

There were no significant differences regarding sleep efficiency (SE) at any of the evaluated times. According to the American Academy of Sleep Medicine (AASM), normal sleep efficiency, utilized as a parameter for the diagnosis of possible sleep disorders, should be above 85% [37]. In this study, however, it was observed in all groups that the overall mean SE on working days was always below the lower limit of 85% recommended by the ASSM.

It was observed in this study that all workers reported feeling sleepy (ESS scores), with the highest proportion in G3-*S* (85.71%). It should be highlighted, however, that the perception of sleepiness and tiredness is subjective and can be confused with feelings of fatigue. Furthermore, human fatigue has not yet been satisfactorily defined because its mechanisms and consequences are complex and there are no objective measures for it [38]. Studies that evaluated fatigue in workers using subjective questionnaires showed that this variable directly depends on the time of the shift being evaluated [9,10].

We calculated in this study the risk index for fatigue (RIF). G1-*NC* showed lower RIF values at every moment than did the other groups. G2-*PS* showed no difference compared with G3-*S*. Thus, our data demonstrate that not having children contributes significantly to reducing RIF scores at the beginning and the end of work shifts. In contrast, we observed a significant increase in RIF values at the final moment of work in comparison with the beginning of a shift in all groups. One factor that could influence the risk of fatigue is the type of work performed. In this sense, activities requiring great physical or mental exertion could increase fatigue and the sleep time required for these workers to recover. However, in our sample, the distribution of job functions was similar between groups (machine operator, mechanic, electrician and welder; see Table 1).

In conclusion, this study indicates that night shift workers without children have longer sleep times and reduced risk for fatigue during their night shifts in relation to workers with children who follow the same work schedule. However, the children’s ages did not contribute to any changes in sleep time or fatigue among the workers with children.
